# Brain metastases in the elderly – Impact of residual tumor volume on overall survival

**DOI:** 10.3389/fonc.2023.1149628

**Published:** 2023-04-04

**Authors:** Lea Baumgart, Amir Kaywan Aftahy, Aida Anetsberger, Dennis Thunstedt, Benedikt Wiestler, Denise Bernhardt, Stephanie E. Combs, Bernhard Meyer, Hanno S. Meyer, Jens Gempt

**Affiliations:** ^1^ Department of Neurosurgery, School of Medicine, Klinikum rechts der Isar, Technical University Munich, Munich, Germany; ^2^ Department of Neurosurgery, University Medical Center Hamburg-Eppendorf, Hamburg, Germany; ^3^ Faculty of Interdisciplinary Studies, University of Applied Sciences Landshut, Landshut, Germany; ^4^ Department of Anesthesiology, School of Medicine, Klinikum rechts der Isar, Technical University Munich, Munich, Germany; ^5^ Department of Neurology, Ludwig Maximilian University (LMU), Munich, Germany; ^6^ Department of Neuroradiology, School of Medicine, Klinikum rechts der Isar, Technical University Munich, Munich, Germany; ^7^ Department of Radiation Oncology, School of Medicine, Klinikum rechts der Isar, Technical University Munich, Munich, Germany; ^8^ German Cancer Consortium (DKTK), Partner Site Munich, Munich, Germany; ^9^ Department of Radiation Sciences (DRS) Helmholtz Zentrum Munich, Institute of Innovative Radiotherapy (iRT), Munich, Germany

**Keywords:** brain metastasis, elderly, overall survival, extent of resection, postoperative MRI, postoperative tumor volume

## Abstract

**Background:**

Due to demographic changes and an increased incidence of cancer with age, the number of patients with brain metastases (BMs) constantly increases, especially among the elderly. Novel systemic therapies, such as immunotherapy, have led to improved survival in recent years, but intracranial tumor progression may occur independently of a systemically effective therapy. Despite the growing number of geriatric patients, they are often overlooked in clinical trials, and there is no consensus on the impact of BM resection on survival.

**Objectives:**

The aim of this study was to analyze the impact of resection and residual tumor volume on clinical outcome and overall survival (OS) in elderly patients suffering from BM.

**Methods:**

Patients ≥ 75 years who had surgery for BM between April 2007 and January 2020 were retrospectively included. Residual tumor burden (RTB) was determined by segmentation of early postoperative brain MRI (72 h). Contrast-enhancing tumor subvolumes were segmented manually. “Postoperative tumor volume” refers to the targeted BMs. Impact of preoperative Karnofsky performance status scale (KPSS), age, sex and RTB on OS was analyzed. Survival analyses were performed using Kaplan-Meier estimates for the univariate analysis and the Cox regression proportional hazards model for the multivariate analysis.

**Results:**

One hundred and one patients were included. Median age at surgery was 78 years (IQR 76-81). Sixty-two patients (61%) had a single BM; 16 patients (16%) had two BMs; 13 patients (13%) had three BMs; and 10 patients (10%) had more than three BMs. Median preoperative tumor burden was 10.3 cm^3^ (IQR 5–25 cm^3^), and postoperative tumor burden was 0 cm^3^ (IQR 0–1.1 cm^3^). Complete cytoreduction (RTB = 0) was achieved in 52 patients (52%). Complete resection of the targeted metastases was achieved in 78 patients (78%). Median OS was 7 months (IQR 2–11). In univariate analysis, high preoperative KPSS (HR 0.986, 95% CI 0.973–0.998, p = 0.026) and small postoperative tumor burden (HR 1.025, 95% CI 1.002–1.047, p = 0.029) were significantly associated with prolonged OS. Patients with RTB = 0 survived significantly longer than those with residual tumor did (12 [IQR 5–19] vs. 5 [IQR 3–7] months, p = 0.007). Furthermore, prolongation of survival was significantly associated with surgery in patients with favorable KPSS, with an adjusted HR of 0.986 (p = 0.026). However, there were no significances regarding age.

**Conclusions:**

RTB is a strong predictor for prolonged OS, regardless of age or cancer type. Postoperative MRI should confirm the extent of resection, as intraoperative estimates do not warrant a complete resection. It is crucial to aim for maximal cytoreduction to achieve the best long-term outcomes for these patients, despite the fact the patients are advanced in age.

## 1 Introduction

Brain metastases (BMs) occur in 20–30% of patients with systemic cancer and are the most common brain tumor, with high recurrence rates of 40–60% (1–5). In addition, the incidence of newly diagnosed BMs is 3–10 times that of newly diagnosed malignant primary brain tumors (3). In light of an aging population and an increased incidence of cancer with age, the number of patients with BMs is growing, especially elderly patients (2, 6–8). Even though there has been tremendous progress in the field of systemic therapy, particularly in immunotherapy in recent years, allowing patients to survive much longer, intracranial tumor progression may occur despite a systematically effective therapy. However, as advanced age and decreased functional independence are associated with poor outcome in patients with BMs, age and functional status have become key criteria of established classification systems for selecting patients for surgical and adjuvant treatment (1, 9–12). This is critical because elderly patients are a vulnerable group, which is often overlooked in numerous studies. Therefore, the development and provision of optimal treatment options have not been satisfactorily defined to date and remain unclear (6, 7, 10, 12–14). Furthermore, studies demonstrated that surgical resections of BMs have a favorable impact on the pre- and postoperative Karnofsky performance status scale (KPSS), which may also apply to elderly patients (9). For this reason, the impact of surgical treatment on geriatric patients with BM, focusing on postoperative clinical outcome and overall survival, was analyzed in this study.

## 2 Materials and methods

### 2.1 Ethics approval

The local ethics committee approved the study (no. 5626:12). The study was conducted in accordance with the ethical standards of the 1964 Declaration of Helsinki. The ethics committee waived written informed consent.

### 2.2 Patient collective

A retrospective chart of elderly patients (defined as age 75 and older) with BMs was developed. Between April 2007 and January 2020, the department surgically treated 119 patients (≥ 75 years) with newly diagnosed single or multiple BMs. Eighteen patients (15%) had biopsies only and did not receive postoperative MRIs. One hundred and one patients (85%) met the inclusion criteria (i.e., histopathological diagnosis of a BM; availability of preoperative MRI and early postoperative MRI (within 72 hours after surgery), and tumor resection beyond biopsy). Key demographic variables, including age at diagnosis, sex, smoking status, number of BMs, tumor localization, pre- and postoperative KPSS, and pre- and postoperative tumor volumes related to the targeted BMs, were evaluated. Furthermore, postoperative treatment structure (radiotherapy), and date of death or date of last contact were reviewed. Progression free survival (PFS), and overall survival (OS) were analyzed.

### 2.3 Surgery

Surgery was performed with the goal of maximum tumor resection while sparing eloquent regions of the brain. Intraoperative neuronavigation was routinely used. If necessary, neuromonitoring and preoperative mapping were also conducted. The indication for surgical treatment was based on the decision of an interdisciplinary neuro-oncology tumor board. The decision was usually based on the following parameters: (1) symptomatic “target” lesion, (2) mass effect, (3) intratumoral hemorrhage, (4) unclear diagnosis, and (5) large posterior fossa tumors with consecutive risk of herniation and hydrocephalus. Intraoperative frozen sections were obtained in all patients.

### 2.4 Residual tumor volume

Within 72 hours after surgery, all postoperative MRIs were analyzed, and postoperative tumor volume was obtained. T1-weighted MRI sequences with gadolinium contrast media were investigated. Any remaining contrast-enhancing lesions were classified as residual tumor, including those that measured less than 10 mm in at least one dimension of imaging. “Postoperative tumor volume” refers to the targeted BMs. Moreover, the term of complete resection also refers to the targeted BMs, whereas complete cytoreduction (RTB=0) addresses all BMs. An experienced neuroradiologist (BW, 10 years of experience) and neurosurgeon (KA, 6 years of experience) performed volumetric measurements. Volumes of the contrast-enhancing tumor parts were manually segmented using the Origin^®^software (Origin^®^, Brainlab, version 3.1, Brainlab AG, Munich, Germany).

### 2.5 KPSS and postoperative treatment structure

KPSS was used to classify and quantify the patient’s pre- and postoperative functional status. The performance status was rated on a numerical scale ranging from 0 to 100, representing the patient’s ability to conduct normal activity, to undertake active work, and need for assistance, with 100 representing full activity and 0 representing death. Adjuvant therapy was selected on an individual basis for each patient after histological diagnosis by an interdisciplinary tumor board. Adjuvant radiation recommendations were based on a variety of factors, including the number of brain lesions, extent of resection (EOR), and the KPSS. Follow-up data was gathered from the institutional outpatient clinic’s electronic patient files, as well as paper-based correspondence from the treating oncologists.

### 2.6 Statistical analysis

Data that have a normal distribution are expressed as mean ± standard deviation (SD) and non-normally distributed data as median and interquartile range (IQR). Logistic regression analyses were performed to identify possible risk factors for outcome changes. Survival analyses were performed using Kaplan-Meier estimates for the univariate analysis and Cox regression proportional hazards model for the multivariate analysis. To determine the optimal cutoff for differences in survival curves, the maximally selected log-rank statistic was found, followed by a comparison of the survival curves separated by the resulting cutoff. A two-tailed significance level of p < 0.05 was defined as statistically significant.

## 3 Results

### 3.1 Patient population

**Figure 1:**
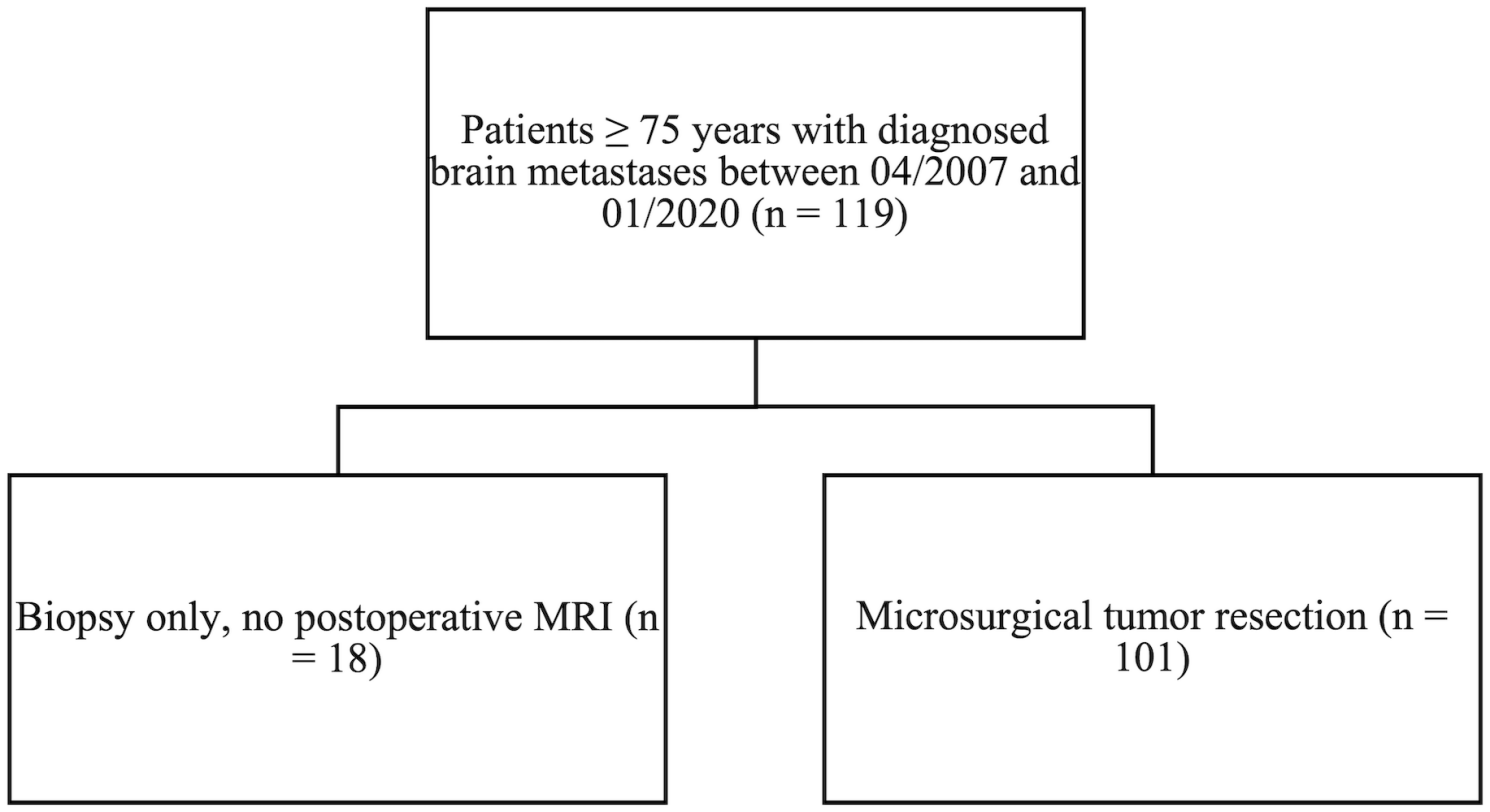
All patients ≥ 75 years with diagnosed brain metastases between April 2007 and January 2020. Patients who met the inclusion criteria of tumor resection beyond biopsy.

One hundred and one patients (85%) met the inclusion criteria (see Methods, **Figure 1**); 56% were male. Median age at surgery was 78 years (IQR 76-81 years). Elderly BM patients presented with a median preoperative KPSS of 80% (IQR 60–90). Median postoperative KPSS was 70% (IQR 60–90). Sixty-two patients (61%) had a single BM; 16 patients (16%) had two BMs; 13 patients (13%) had 3 BMs; and 10 patients (10%) had three or more BMs. BMs most commonly originated from cancers of the lung (23%), melanoma (22%), and breast (11%). Median preoperative tumor burden of the targeted lesions was 10.3 cm^3^ (IQR 5–25 cm^3^) and postoperative tumor burden 0 cm^3^ (IQR 0–1.1 cm^3^). Seventy-four patients (73%) underwent postoperative radiotherapy; about half of those (46%) had HSRS; 38% had whole brain radiation therapy (WBRT); and 3% had SRS (**Table 1**). For 13% of patients, information on radiation modality was unavailable. Twenty-three patients (23%) underwent postoperative chemotherapy, and 14 patients (14%) had immunotherapy.

**Table 1:** Baseline characteristics of included patients, tumor data, and postoperative therapy.

Demographics N (%) or median (range/IQR)	Included patients (n = 101)
Sex	F 44/101 (44) M 57/101 (56)
Age	78 (IQR 76–81)
Karnofsky Performance Status Scale (KPSS)	
Preoperative KPSS	80% (IQR 60–90)
Postoperative KPSS	70% (IQR 60–90)
Number of metastases N (%)	
1	62 (61)
2	16 (16)
3	13 (13)
> 3	10 (10)
Postoperative radiotherapy N (%)	
none	27 (27)
WBRT	28 (28)
SRS	2 (2)
HSRS	34 (34)
Tumor burden (cm^3^) median (IQR)	
Preoperative	10.3 cm^3^ (5–25 cm^3^)
Postoperative	0 cm^3^ (0–1.1 cm^3^)
Primary tumor N (%)	
NSCLC	21 (21)
SCLC	2 (2)
Melanoma	22 (22)
Breast	11 (11)
Carcinoma of unknown primary	10 (10)
Prostate	9 (9)
Colon/Rectum	7 (7)
Renal cell carcinoma	6 (6)
Transitional cell carcinoma	3 (3)
Gynecologic	2 (2)
Thyroid	2 (2)
Stomach	2 (2)
Other	4 (4)

### 3.2 Impact of KPSS and age

According to the univariate analysis, a favorable preoperative KPSS was significantly associated with prolonged survival in this population, with an adjusted HR of 0.986 (p = 0.026). However, the analysis showed age to be a negligible parameter regarding OS (HR 1.024, p = 0.570).

### 3.3 Survival analysis and impact of residual tumor volume

**Figure 2:**
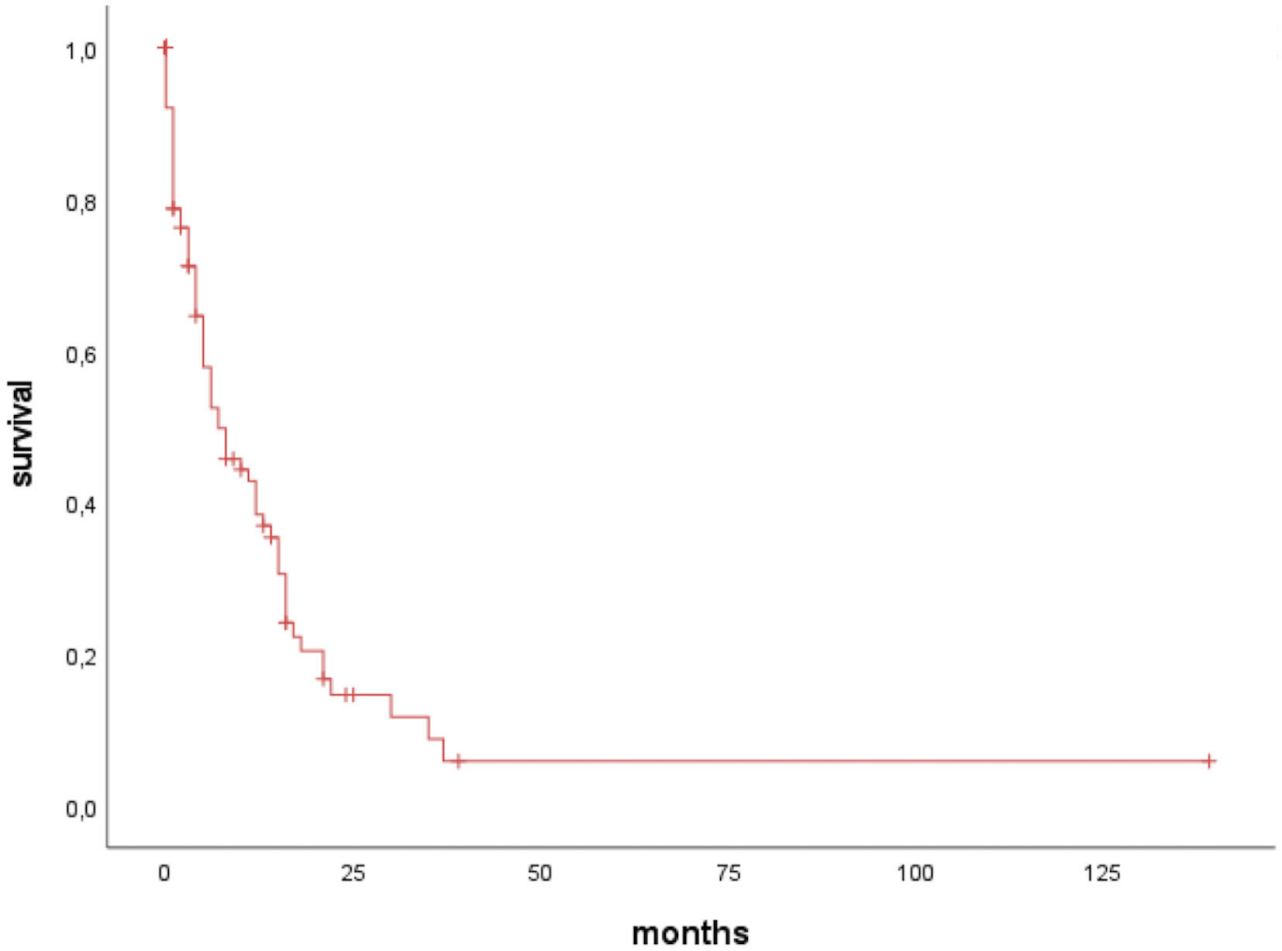
Median overall survival time of all patients.

Median OS of all patients was 7 months (IQR 2–11; **Figure 2**). However, 20% of patients with BMs survived longer than 12 months, and 10% survived longer than 24 months.

**Figure 3:**
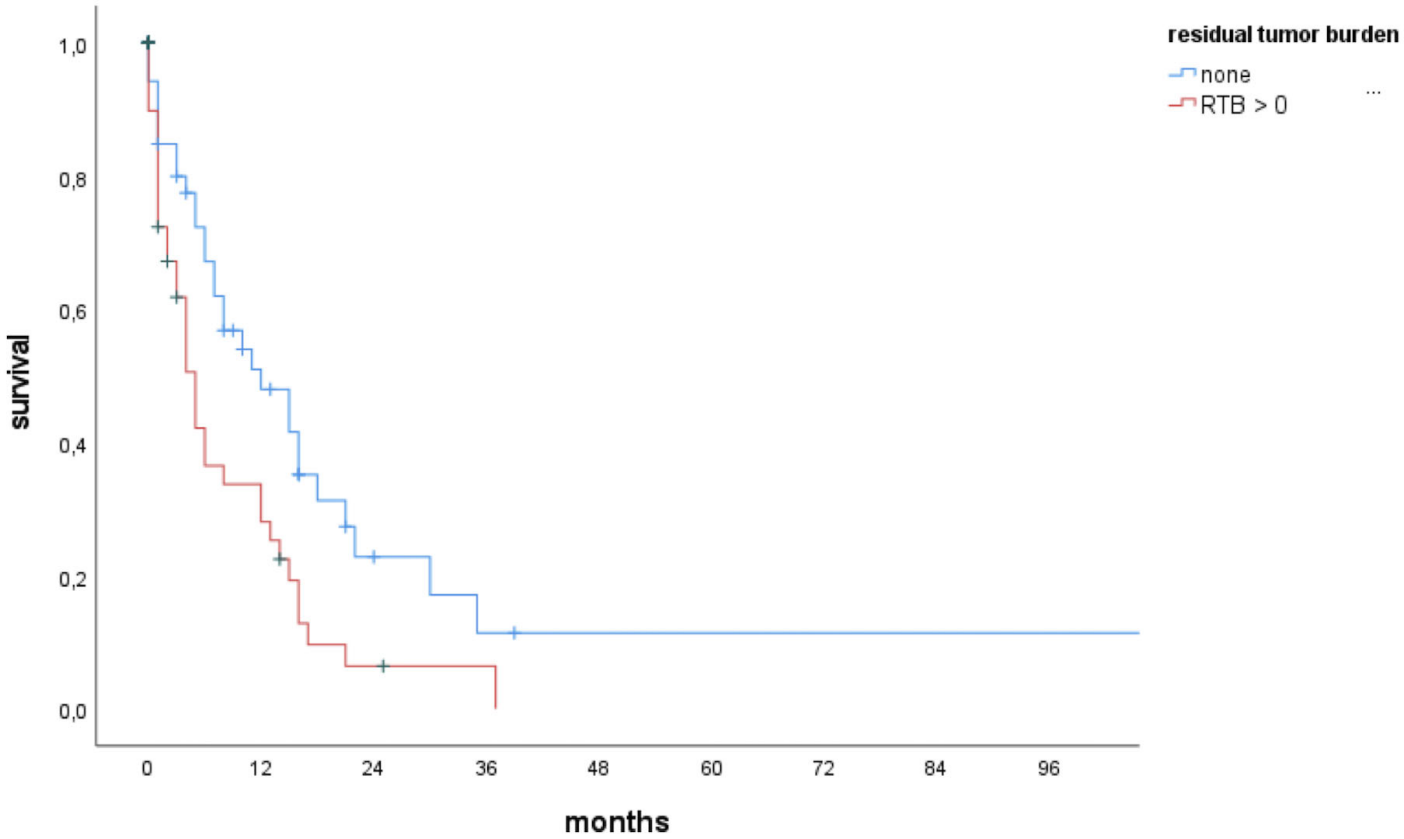
Kaplan-Meier curves for OS of elderly patients with BMs, stratified according to RTB = 0, RTB >0.

Patients with RTB = 0 had significantly higher survival rates (p = 0.007) and longer median survival: 12 (IQR 5–19) versus 5 (IQR 3–7) months, as shown by Kaplan-Meier estimates (**Figure 3**).

A multivariate Cox regression analysis was conducted, including the following risk factors for mortality: sex (female), age, KPSS at admission, preoperative tumor volume, and RTB > 0 **(** **Table 2**). Cox regression multivariate analysis identified RTB > 0 to be a significant risk factor for shorter OS (HR 1.898, IQR 1.162–3.099, p = 0.010).

**Table 2:** Multivariate Cox regression analysis including the following risk factors for mortality: sex (female), age, KPSS at admission, preoperative tumor volume, and RTB > 0.

Multivariate analysis parameters	Hazard Ratio (IQR)	p-value
Sex (female)	1.361 (0.819-2.262)	0.235
Age	1.040 (0.956-1.130)	0.363
KPSS at admission	0.992 (0.978-1.006)	0.260
Preoperative Tumor volume	1.009 (0.998-1.021)	0.118
RTB > 0	1.898 (1.162–3.099)	0.010

In a subgroup analysis, patients who underwent complete resection of the targeted metastasis, but had intracranial tumor burden at other locations (N = 78), still had significantly higher survival rates than patients with incomplete resection of the targeted BM (p = 0.042) did. In addition, these patients had longer median OS: 8 (IQR 3–13) versus 5 (IQR 2–8) months.

Patients who underwent postoperative radiotherapy did not have significantly longer OS than the patients who did not receive postoperative radiotherapy.

### 3.4 Patients with single brain metastasis

**Figure 4:**
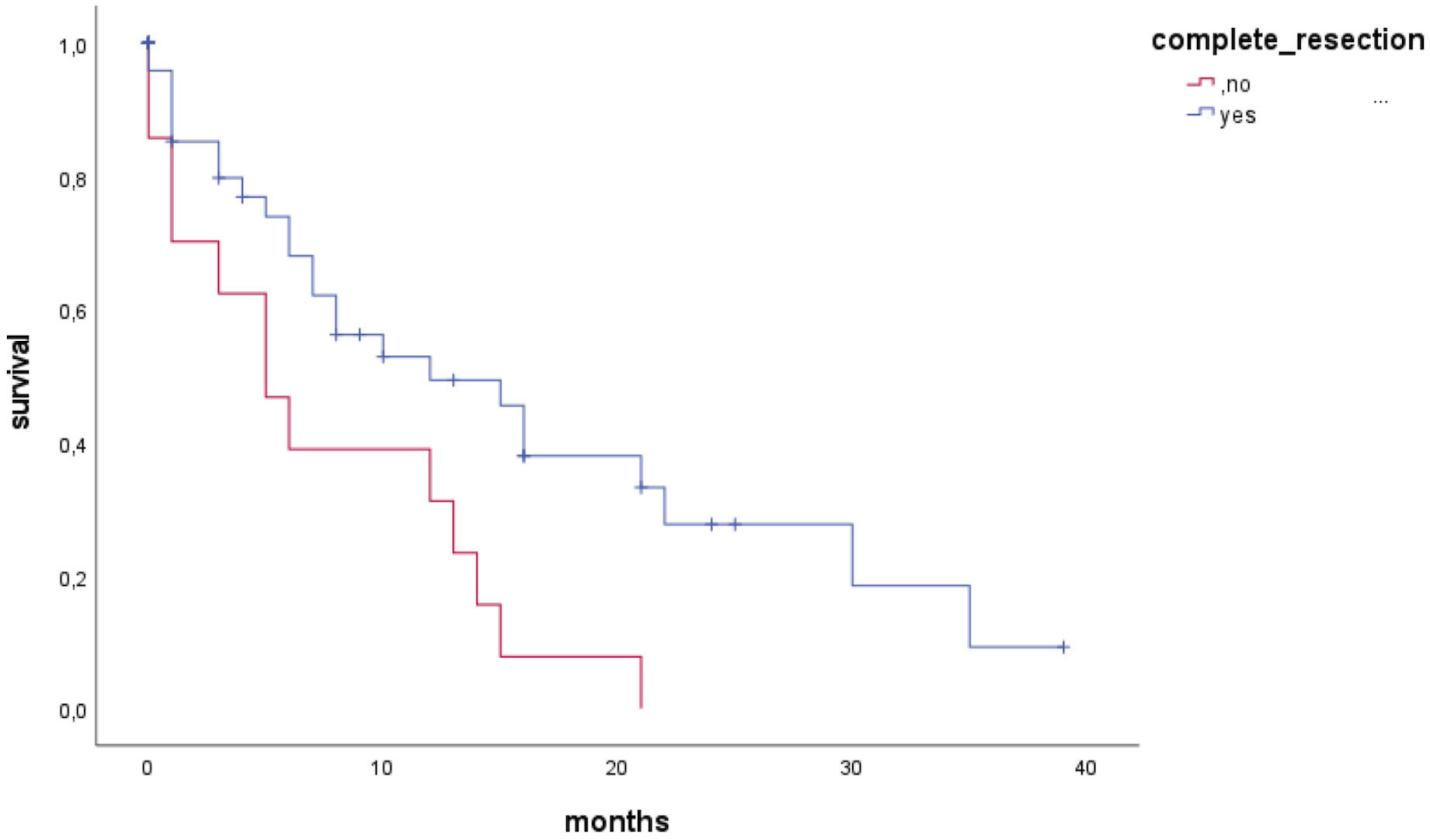
Kaplan-Meier curves for OS of elderly patients with a single brain metastasis (singular, solitary), stratified for complete vs. incomplete resection.

Considering only patients with a single BM (n = 62), 48 patients (77%) had complete resection. Cox regression multivariate analysis including the above-mentioned risk factors showed incomplete resection a significant risk factor for mortality (HR = 2.398, IQR 1.198-4.779, p = 0.011). Therefore, patients with complete resection have a significantly higher OS (**Figure 4**).

## 4 Discussion

The results clearly indicate that, for elderly patients with BMs, surgical resection of BMs significantly improves PFS and OS, as RTB is a strong predictor for OS, regardless of age or cancer type. Preoperative high KPSS (HR 0.986, 95% CI 0.973–0.998, p = 0.026) and low RTB (HR 1.025, 95% CI 1.002–1.047, p = 0.029) were significant protective factors for longer OS. Hereby, RTB = 0 was significantly associated with prolonged median OS compared to RTB > 0 (12 months vs. 5 months, p = 0.007). Furthermore, prolongation of survival was significantly associated with surgery in patients who had a favorable KPSS (adjusted HR of 0.986, p = 0.026). However, there were no significances regarding age.

### 4.1 Impact of age and KPSS

Several studies have found that lower age is a protective factor for prolonged overall survival in patients receiving surgical BM resections (15, 16). This study’s data does not support this, as younger age did not correlate with lower postoperative complication rates or better outcomes. High preoperative KPSS was found to be a significant parameter for prolonged OS. KPSS is a critical parameter when choosing adjuvant therapy (5, 17–20). The reason for the statistical results regarding age in this study is the highly age-selected patient population, as only patients ≥ 75 years were included in the study. Thus, biological age (KPSS) plays a more decisive role, than calendar age does, in patients who have exceeded a certain age.

Therefore, a crucial outcome can be stated, which is beneficial for future treatment of elderly patients with BMs. High KPSS is the more important protective prognostic factor, for better neurological outcomes and longer OS. Thus, when looking at the postoperative treatment structure, patients with a high KPSS and good functional status are more likely to receive adjuvant therapy, which is critical for a long PFS and OS (5, 17, 18, 21).

As the cut-off age of ≤ 65 years in recent publications often overlooks elderly patients, the findings are even more meaningful (6, 7, 9, 12, 14). Advanced age was often determined to be a poor prognostic factor (15). Therefore, surgery for patients in this age group was only performed with caution.

This study showed that advanced age was a negligible risk factor in the selected cohort.

### 4.2 Survival analysis and impact of residual tumor volume

EOR has been a focus of research on the treatment of older glioma patients, and cytoreduction has been shown to improve overall survival in the elderly (20, 22). This study shows that maximal EOR is critical for good long-term outcomes in older patients with BMs, as RTB = 0 significantly improves OS and local tumor control regardless of the cancer type. This finding is in accordance with recent investigations regarding the impact of RTB in younger patients (≤ 65 years) with brain metastases, which have proven low RTB to be a significant predictor for prolonged OS (23). Furthermore, it has been argued that maximal cytoreduction should be achieved, independent of the postoperative radiotherapy type (23). Postoperative MRI should confirm EOR, as intraoperative estimates can misjudge complete resection. Furthermore, previous analyses identified EOR as a strong prognostic factor for long-term local tumor control (13).

Radiotherapy and systemic therapy are important components in oncological therapy of BMs. Data from prospective randomized trials demonstrate that radiotherapy has significant impact on local tumor control and OS (24–26). However, in this research, postoperative radiotherapy did not have a significant impact on prolonged OS. This result can be interpreted by the assumption that, in the selected cohort, only a small proportion of patients did not receive postoperative radiotherapy. To identify the effect of radiotherapy more clearly, the number of patients in the investigated cohort would need to be greater. Furthermore, due to the highly selected patient population in this investigation, there may be a slight bias regarding these results.

Nevertheless, in this study and as shown by Sivansaker et al., the EOR was significantly more decisive for local tumor control and patients’ prolonged OS (13). Thus, detailed analysis focusing on postoperative treatment options should be considered in future interdisciplinary studies.

### 4.3 Study limitations

This study offers a single-center experience with a reasonable number of patients, as well as a homogeneous diagnostic and treatment strategy that allows for comparison. However, it has some limitations.

A limitation of this trial is the retrospective study design, as it introduces an unavoidable selection bias. Furthermore, new histopathological and molecular pathological findings have been discovered, and therapeutic options have been dynamically expanded in recent years, resulting in heterogeneity in the current population. This study cannot reflect the most recent innovations and improvements in systematic chemotherapy for the different cancer entities.

## 5 Conclusions

In conclusion, the results indicate that, in elderly patients with BMs, surgical resection of BMs improves PFS and OS, as RTB is a strong predictor for OS, regardless of age or cancer type. Tumor remnant in an early postoperative MRI is the only risk factor for local in-brain recurrence. Therefore, a postoperative MRI should be used to confirm the EOR. Although more patients with metastasized cancer have been treated aggressively in recent years, some studies have argued that elderly patients with brain metastases should not be resected because there might be a higher prevalence of treatment-related toxicity, leading to a poorer prognosis (27). This paradigm must be reconsidered: the data suggests that these patients also benefit significantly from maximal surgical cytoreduction in the presence of intracranial metastases, despite advanced age. The results indicate that, in the elderly, the prognostic value of age becomes less important for outcome and OS, especially among patients ≥75 years. Instead, the patient’s functional status is the most important prognostic factor for long-term outcome and OS. Additionally, surgical resection can enable adjuvant therapy that would otherwise have been impossible by improving the functional outcome.

## Data availability statement

The raw data supporting the conclusions of this article will be made available by the authors, without undue reservation.

## Ethics statement

The local ethics committee approved the study (no. 5626:12). The study was conducted in accordance with the ethical standards of the 1964 Declaration of Helsinki. The ethics committee waived written informed consent.

## Author contributions

Conceptualization, LB and JG. Methodology, LB. Software, LB. Formal analysis, AA. Investigation, LB, AA, BW, AKA. Writing—original draft preparation, LB. Writing—review and editing, LB, AKA, AA, DT, BW, DB, SC, BM, HM, JG. Visualization, AA. Supervision, JG, BM, HM, BW, DB, SC. Project administration, BM and JG. All authors contributed to the article and approved the submitted version.
